# Effects of exercise on kidney and physical function in patients with non-dialysis chronic kidney disease: a systematic review and meta-analysis

**DOI:** 10.1038/s41598-020-75405-x

**Published:** 2020-10-23

**Authors:** Keisuke Nakamura, Tomohiro Sasaki, Shuhei Yamamoto, Hiroto Hayashi, Shinji Ako, Yuu Tanaka

**Affiliations:** 1 Department of Rehabilitation, Matsumoto City Hospital, Nagano, Japan; 2 Department of Rehabilitation, Shinshu University Hospital, Nagano, Japan; 3 Department of Public Health, Graduate School of Medical and Dental Sciences, Tokyo Medical and Dental University, Tokyo, Japan; 4 Department of Internal Medicine, Kidney & dialysis center, Matsumoto City Hospital, Nagano, Japan; 5 Department of Anesthesiology, Nara Medical University, Nara, Japan; 6 Present Address: Department of Rehabilitation, Matsumoto City Hospital, 4417-180 Hata, Matsumoto, Nagano 390-1401 Japan

**Keywords:** Nephrology, Kidney diseases, Metabolic disorders

## Abstract

Patients with non-dialysis chronic kidney disease (CKD) are at greater risk of early mortality and decreased physical function with an advance in the stage of CKD. However, the effect of exercise in these patients is unclear. This meta-analysis aimed to determine the effects of physical exercise training on the risk of mortality, kidney and physical functions, and adverse events in patients with non-dialysis CKD. The meta-analysis conformed to the Preferred Reporting Items for Systematic Reviews and Meta-Analysis (PRISMA) statement and the Cochrane Handbook recommendations. On 16 August 2019, the PubMed, CINAHL, Cochrane Library databases, and Embase were electronically searched, with no restrictions for date/time, language, document type, or publication status, for eligible randomized controlled trials (RCTs) investigating the effects of exercise on mortality and kidney and physical function in patients with non-dialysis CKD. Eighteen trials (28 records), including 848 patients, were analyzed. The effects of exercise on all-cause mortality and estimated glomerular filtration rate were not significantly different from that of usual care. Exercise training improved peak/maximum oxygen consumption compared to usual care. Regular exercise improves physical and walking capacity for patients with non-dialysis CKD. Effect on leg muscle strength was unclear.

## Introduction

Chronic kidney disease (CKD) is a major clinical condition affecting a significant number of individuals worldwide; additionally, it is associated with high-risk cardiovascular disease (CVD), stroke, frailty, and mortality^1–6^. Furthermore, there is a significant association between the severity of CKD and health care costs^7^. For these reasons, optimal management of CKD is especially important to prevent kidney failure, extend healthy life expectancy, and have a positive impact on health care costs.

The primary strategies for the prevention and treatment of CKD include lifestyle changes and pharmacological approaches, including the promotion of exercise, dietary changes, and antihypertensive drugs^2^. Studies have shown that physical function and performance in pre-dialysis CKD decreased with an advance in the stage of CKD, which may be caused by several factors, including decreased kidney function, chronic inflammation, and arteriosclerosis^8–10^. A systematic review reported that improved physical function and greater levels of physical activity in pre-dialysis CKD reduced all-cause and cardiovascular mortality risk^11^; this highlights the important role of exercise interventions for improvement of physical function and activity levels in this population. Several systematic reviews have reported that exercise training, including both aerobic and resistance exercises, has significant positive effects on physical fitness, including physical and functional capacity, muscle strength, and blood pressure in patients with CKD^12–14^. However, these reviews included participants receiving dialysis therapy. There has been no systematic review or meta-analysis evaluating the effects of aerobic and resistance exercise on kidney and physical functions, adverse events, and mortality in patients with non-dialysis CKD. A systematic review conducted in 2014 reported differences in the effect of exercise training on aerobic capacity, muscular functioning, and health-related quality of life that may depend on CKD stage, dialysis treatment, and history of a kidney transplant. However, that systematic review featured only one clinical trial including patients with CKD stages 2–5, and it was impossible to conduct a meta-analysis^13^. Since 2014, there have been various RCTs evaluating the effects of physical exercise on patients with non-dialysis CKD^15,16^. The investigation of the effects of exercise on physical function in patients with non-dialysis CKD is clinically important because there are　differences between dialysis and non-dialysis patients, including glomerular filtration rate (GFR), risk of mortality and lifestyle habits^2,3^.

Therefore, this systematic review and meta-analysis aimed to determine the effects of physical exercise training on risk of mortality, kidney function, adverse events, and physical function outcomes [i.e., exercise tolerance (peak/maximum oxygen consumption [VO_2_]), walking ability (6-min walk distance), and lower extremity muscle strength] in adult patients with non-dialysis CKD.

## Results

**Figure 1:**
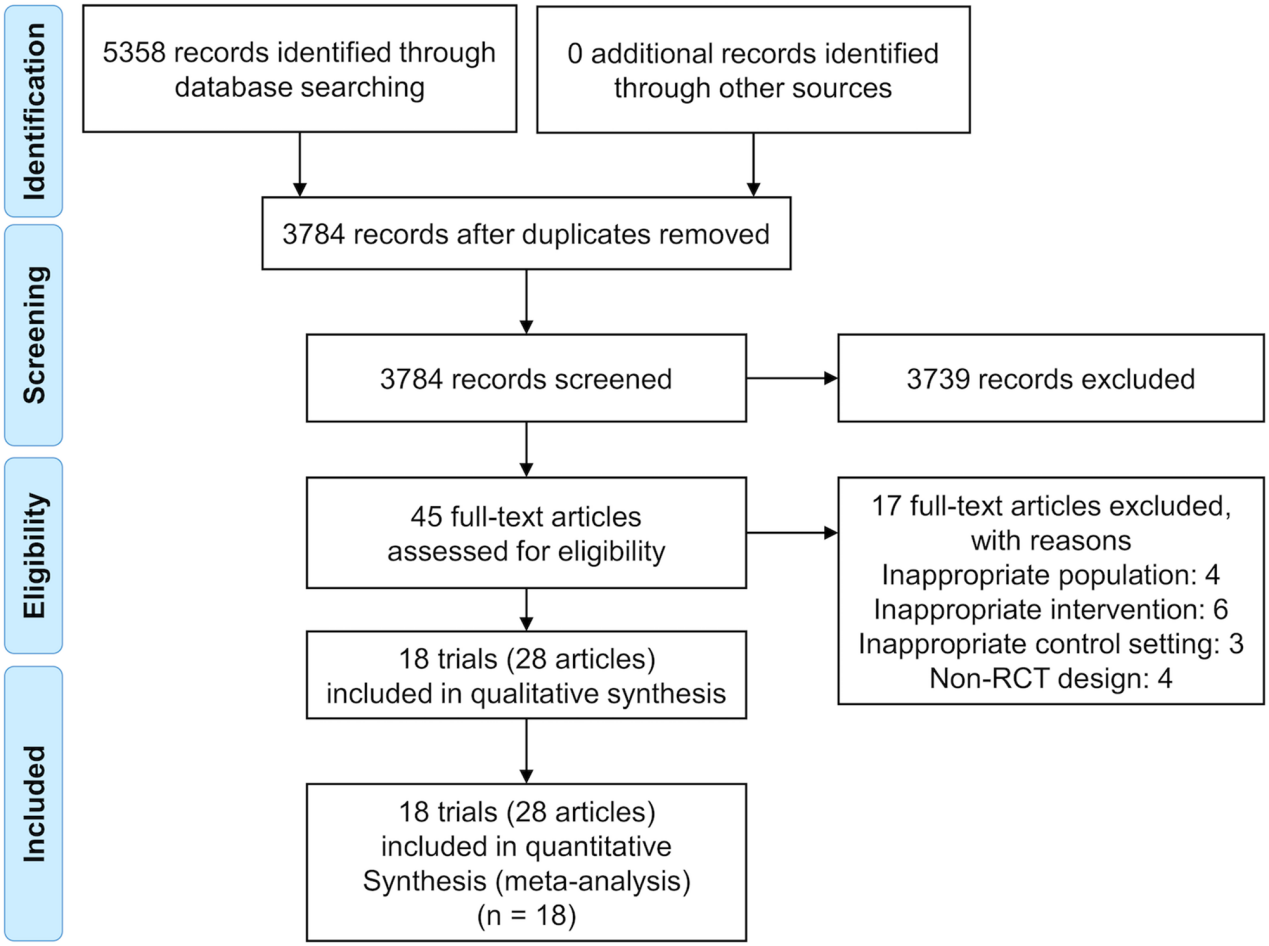
Study flow diagram.

In total, 3784 records were identified after the removal of duplicates, and 45 records remained after the screening of titles and abstracts. Further, 17 records were excluded based on the full eligibility criteria. In total, 18 trials (28 records)^8,15,17–42^ including 848 patients with non-dialysis CKD who had met the eligibility criteria of this review were included in the analysis (Fig. 1). The characteristics of the trials included in this review are described in Table 1. Overall, the records demonstrated a broad range of follow-up duration (median follow-up = 20.5 weeks; range 8−72 weeks) and CKD stage of the trial population (CKD stages 3–4, 9 trials; stages 2–4, 4 trials; stage 3, 1 trial; stages 3–5, 1 trial; stages 1–3, 1 trial; not reported, 2 trials). Categorizations of the types of exercise training in the trials were as follows: center-based exercise = 9 trials, home-based exercise = 4 trials, combined both center and home-based exercise = 6 trials, aerobic exercise = 8 trials, resistance exercise = 2 trials, and combined both aerobic and resistance training = 8 trials.

**Table 1:** Characteristics of the included studies.

Trials	N (analyzed Exp/Con)	Participants					Control group	Intervention group	Compliance of intervention group (%)	Outcome measures	Follow-up assessment
		Age (years)	BMI	CKD stage	eGFR (mL/min/1.73 m^2^)	Percentage of DM (%)					
Kirkman^17^ 2019, USA	16/15	58	32	Stage 3–5	44	ND	Routine care	Centre-based aerobic exercise for 12 weeks Type: cycling, walking, jogging, elliptical machine Frequency: 3 times/week Intensity: 60–85% HRR, RPE 12–16 Duration: 45 min	92	VO_2_ peak, eGFR	12 weeks
Aoike^18^ 2018, Brazil (Aoike^31^ 2015, Baria^33^ 2014, Gomes^24^ 2017)	25/15	55.8	31.2	Stage 3–4	26.9	35	Usual care	Centre- and home-based aerobic exercise for 24 weeks Type: Walking or treadmill Frequency: 3 times/w Intensity: the heart rate value obtained at VT Duration: for 30 min with increments of 10 min in duration every 4 weeks until week 8	ND	VO_2_ peak, eGFR, Cr, 6MWT	24 weeks
Barcellos^20^ 2018, Brazil	58/51	65	29.9	Stage 2–4	62.6	0	Usual care	Centre-based aerobic and resistance exercises for 16 weeks Type: unclear Frequency: 3 times/week Intensity: unclear Duration: unclear	63.7	eGFR, TUG	16 weeks
Beetham^19^ 2018, Australia (Howden^15,35^ 2013, 2015, Small^26^ 2017)	74/68	63.5	33.1	Stage 3–4	40.5	42.2	Usual care	Centre-based aerobic and resistance exercises for 8 weeks, followed by home-based aerobic and resistance exercise for 10 months Type: Aerobic, treadmill, stationary bike, rowing ergometer Resistance, machine, free weight Frequency: 2–3 times/week Intensity: moderate intensity, with perceived exertion of 11–13 on the 20-point Borg scale Duration: 150 min per week	ND	VO_2_peak, eGFR, Cr, 6MWT, TUG	52 weeks
Ikizler^21^ 2018, USA	46/46	60	33	Stage 3–4	41	25	Usual physical activity + usual diet	Centre-based aerobic exercises for 4 months Type: Aerobic; a treadmill, an elliptical cross trainer, a Nu–Step cross trainer, and a recumbent stationary bicycle Frequency: 3 times/week Intensity: 60–80% VO_2_ max Duration: 30–45 min	85	VO_2_ peak, eGFR, Cr	17 weeks
Hiraki^23^ 2017, Japan	14/14	68.7	23.7	Stage 3–4	39	7.1	Usual care	Home-based aerobic and resistance exercises for 1 year Type: Aerobic: brisk walking Resistance: handgrip strengthening devise, squat, calf raise Frequency: 3 times/week Intensity: midlevel intensity, with perceived exertion on the Borg scale Duration: 30 min or completing 8000–10,000 steps/day	70.4	Leg muscle strength, eGFR	52 weeks
Leehey^16^ 2016, USA	14/18	66	36.8	Stage 2–4	40	100	Only a nutritional counseling	Centre-based aerobic and resistance exercises for 12 weeks followed by 40 weeks of home-based aerobic and resistance exercises (total duration of study 52 weeks) Type: Aerobic, interval training on a treadmill; Resistance, elastic bands, handheld weights or weight machine Frequency: 3 times/w Intensity: Aerobic, almost moderate (> 50% of total time), and the rest was light or hard intensity; Resistance, none stated Duration: Centre-based, 60 min of aerobic and 20–30 min of resistance training. Home-based, 60 min trice weekly or 30 min 6 times a week	ND	VO_2_ peak, eGFR, Cr, 6MWT, TUG, leg muscle strength	52 weeks
Tang^27^ 2016, China	42/42	45.1	23.6	Stage 1–3	ND	ND	Usual care	Home-based aerobic exercises for 12 weeks Type: Aerobic, walking, cycling, jogging Frequency: 3 times/week Intensity: Moderate intensity, with perceived exertion of 12–15 on the 20-point Borg scale Duration: 20–30 min	ND	6MWT	12 weeks
Greenwood^28^ 2015, UK	8/10	53.5	28	Stage 3–4	42.1	11.1	Usual care	Centre- and home-based aerobic and resistance exercise for 12 months Type: Aerobic, cycling; Resistance, weight machine Frequency: 3 times/week Intensity: Aerobic, 80% HR reserve with maximum heart rate; Resistance, 80% of 1RM Duration: Aerobic, two 20-min sessions and eventually one 40-min session Resistance: 3 sets × 10 repetitions	79.2	VO_2_peak, eGFR, Cr	52 weeks
Van Craenenbroeck^30^ 2015, Belgium	19/21	53.2	28.3	Stage 3–4	38.6	7.5	Standard therapy without specific instructions about physical activity	Centre- and home-based aerobic exercise for 12 weeks Type: Aerobic, cycling Frequency: In the first 2 weeks of the study period, at least 3 training sessions were supervised in the hospital by an experienced medical doctor. For the following 2 weeks, a supervised training session was organized once a week.70 or more training days for 12 weeks Intensity: 90% of the heart rate achieved at the anaerobic threshold on baseline testing Duration: 4 × 10 min	95.4	VO_2_ peak, eGFR	12 weeks
Watson 2015^29^, UK	18/15	Exp:63/Con:66*	32.2	Stage 3b-4	Exp:28.5/Con:20.5*	Exp:15/Con:27	Usual activity	Centre-based resistance exercise for 8 weeks Type: resistance machine Frequency: 3 times/w Intensity: 70% of 1RM Duration: 3 sets × 10–12 repetitions	92	Leg muscle strength	8 weeks
Headley^34^ 2014, USA (Headley^22^ 2017, Miele^25^ 2017)	25/21	57.6	35.6	Stage 3	47.6	4.3	Usual care	Center–based aerobic and resistance exercises for 16 weeks Type: treadmill, brisk walking Frequency: 3 times/ week Intensity: 50% – 60% of the VO_2_ peak Duration: 45 min	96.9	VO_2_ peak	16 weeks
Rossi^32^ 2014, USA	48/46	68.5	31.5	Stage 3–4	ND	41.1	Only standard CKD clinic care	Center–based aerobic and resistance exercise for 12 weeks Type: Aerobic; treadmill walking and/or stationary cycling Resistance; free weights (upper and lower extremity) Frequency: 2 times/week Intensity: Aerobic, a RPE corresponding to a 60%–65% predicted maximal heart rate. Resistance, using 1–10-lb. weights (according to tolerance) Duration: Aerobic, 60 min; Resistance, three sets of 15 repetitions	72.9	6MWT	12 weeks
Headley^36^ 2012, USA (Gregory^37^ 2011)	10/11	54.9	33.5	Stage 2–4	41.2	33.3	Standard of care	Centre-based aerobic and resistance exercises for 48 weeks Type: Aerobic: treadmill, cycle ergometer, elliptical machines, Stairmaster Resistance: machine weight Frequency: Aerobic, 3 times per week; Resistance, 2 times per week Intensity: 50%–60% of the VO_2_ peak Duration: Aerobic, 45 min; Resistance, two sets of 10–15 repetitions	83.8	VO_2_ peak, eGFR	48 weeks
Mustata^38^ 2011, Canada	10/10	Exp:64, Con:72.5*	28.3	Stage 3–4	Exp: 27.0, Con:28.0*	55	Standard care	Centre- and home-based aerobic exercises for 12 months Type: Treadmill, cycle ergometer, elliptical machines, walking Frequency: Centre, 2 times/week; Home, 3 times/week Intensity: 40%–60% of the VO_2_ peak Duration: 60 min	80	VO_2_ peak, eGFR	52 weeks
Leehey^39^ 2009, USA	7/4	66	ND	Stage 2–4	45.1	100	Standard of care medical treatment for diabetes and CKD	Centre-based aerobic exercises for 6 weeks followed by 18 weeks of home-based aerobic exercises (total duration of study 24 weeks) Type: Treadmill or walking Frequency: 3 times/w Intensity: Almost moderate (> 50% of total time), and the rest was light or hard intensity Duration: Center; 40 min Home; increase their step count/structured walk by 10% each week	ND	VO_2_ max, Cr	24 weeks
Castaneda^41^ 2001, USA (Castaneda^40^ 2001)	14/12	Exp:65/Con:64	28.1	ND	26	38	A low–protein diet plus sham exercises	A low–protein diet plus Center–based resistance training Type: machine Frequency: 3 times/w Intensity: 80% Duration: 45 min	91	Leg muscle strength, eGFR, Cr	12 weeks
Eidemak^42^ 1997, Denmark	15/15	Exp:42/Con:44*	ND	ND	Exp:26/Con:24*	0	Usual care	Home-based aerobic training All patients were followed for a minimum of 1.5 years or until the need for dialysis or kidney transplantation Type: bicycle ergometer, and running, swimming and walking Frequency: every day Intensity: 60–75% VO_2_ max Duration: 30 min of bicycling daily or an equal amount of other physical activities	ND	eGFR (^51^Cr–EDTA), VO_2_ max	A minimum of 78 weeks or until need of dialysis or kidney transplantation

Values of Age, eGFR were expressed as mean.

* median, *ND* no data, *VO*_*2*_ oxygen uptakes, *eGFR* estimated glomerular filtration rate, *Cr* creatinine, *6MWT* six-minute walk test, *TUG* timed up and go test, *HRR* heart rate reserve, *RPE* rating of perceived exertion, *DM* diabetes mellitus, *RM* repetition maximum.

### Quality assessment

The results of the risk of bias assessment in all the trials are summarized in Table 2. All participants were classified into the exercise training and the usual care groups. Blinding of participants was not possible due to the nature of exercise training. Three trials^17,32,36^ showed high risks of bias related to the predicted direction of bias due to missing outcome data. Only 1 trial^42^ showed different time points of measurements in the outcome data because all patients were followed for a minimum of 1.5 years or until the necessity for dialysis or kidney transplantation, which were possibilities of measurement bias. Seven trials^16,18,19,21,30,32,36^ (within all reported outcomes) had enlisted in clinical trial registries or study protocols, whereas 5 trials^8,17,20,28,41^ had high reporting bias. The clinical trial registries or study protocols of other trials could not be ascertained; thus, reporting bias was unclear.

**Table 2:** Risk of bias summary.

Trials	1. Randomization process	2. Deviations from intended interventions	3. Missing outcome data	4. Measurement of outcome data	5. Selection of the reported results	Overall
Kirkman 2019, USA	Low risk	Some concerns	High risk	Low risk	High risk	High risk
Aoike 2018, Brazil	Low risk	Low risk	Low risk	Low risk	Low risk	Low risk
Barcellos 2018,Brazil	Low risk	Low risk	Some concerns	Low risk	High risk	High risk
Beetham 2018, Australia	Low risk	Low risk	Low risk	Low risk	Low risk	Low risk
Ikizler 2018, USA	Low risk	Low risk	Low risk	Low risk	Low risk	Low risk
Hiraki 2017, Japan	Low risk	Low risk	Low risk	Low risk	High risk	High risk
Leehey 2016, USA	Low risk	Low risk	Some concerns	Low risk	Low risk	Some concerns
Tang 2016, China	Low risk	Low risk	Low risk	Low risk	Some concerns	Some concerns
Greenwood 2015, UK	Some concerns	Low risk	Low risk	Low risk	High risk	High risk
Van Craenenbroeck 2015, Belgium	Low risk	Low risk	Some concerns	Low risk	Low risk	Some concerns
Watson 2015, UK	Low risk	Low risk	Low risk	Low risk	Some concerns	Some concerns
Headley 2014, USA	Low risk	Low risk	Low risk	Low risk	Low risk	Low risk
Rossi 2014, USA	Some concerns	Low risk	High risk	Low risk	Low risk	High risk
Headley 2012, USA	Some concerns	Low risk	High risk	Low risk	Some concerns	High risk
Mustata 2011, Canada	Low risk	Low risk	Low risk	Low risk	Some concerns	Some concerns
Leehey 2009, USA	Low risk	Low risk	Some concerns	Low risk	Some concerns	Some concerns
Castaneda 2001, USA	Some concerns	Low risk	Low risk	Low risk	High risk	High risk
Eidemak 1997, Denmark	Some concerns	Low risk	Low risk	High risk	Some concerns	High risk

### Effects of interventions: primary outcomes

#### All-cause mortality

**Figure 2:**
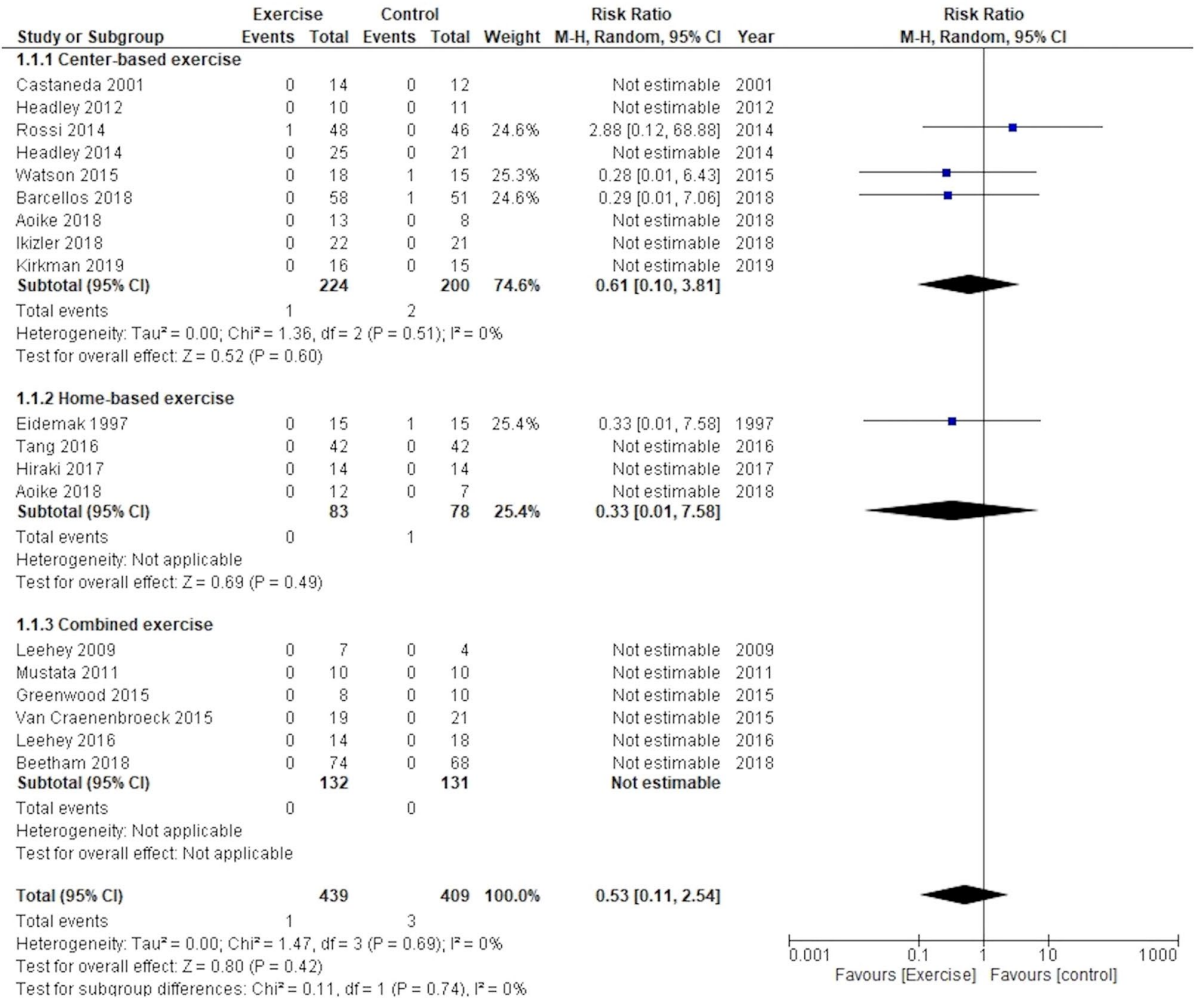
Effect of exercise training on all-cause mortality.

Eighteen trials, including 848 participants, reported all-cause mortality of the follow-up duration. The effect of physical exercise training on all-cause mortality was observed to be uncertain compared to usual care (risk ratio (RR), 0.53; 95% confidence interval (CI), 0.11−2.54; participants = 848, trials = 18; I^2^ = 0%) (Fig. 2). Overall, 4 trials^20,29,32,42^ reported the death of participants; however, those were not related to exercise training. Subgroup analysis performed for exercise types, estimated glomerular filtration rate (eGFR) (< 30 or ≥ 30 mL/min/1.73 m^2^), and body mass index (BMI) (< 30 or ≥ 30 kg/m^2^) showed no evidence of differences between the groups (test for subgroup difference: P = 0.74, 0.49, and 0.68, respectively). Subgroup analysis was not performed for other variables because an I^2^ > 50% was not obtained.

#### Kidney function (eGFR, Scr)

**Figure 3:**
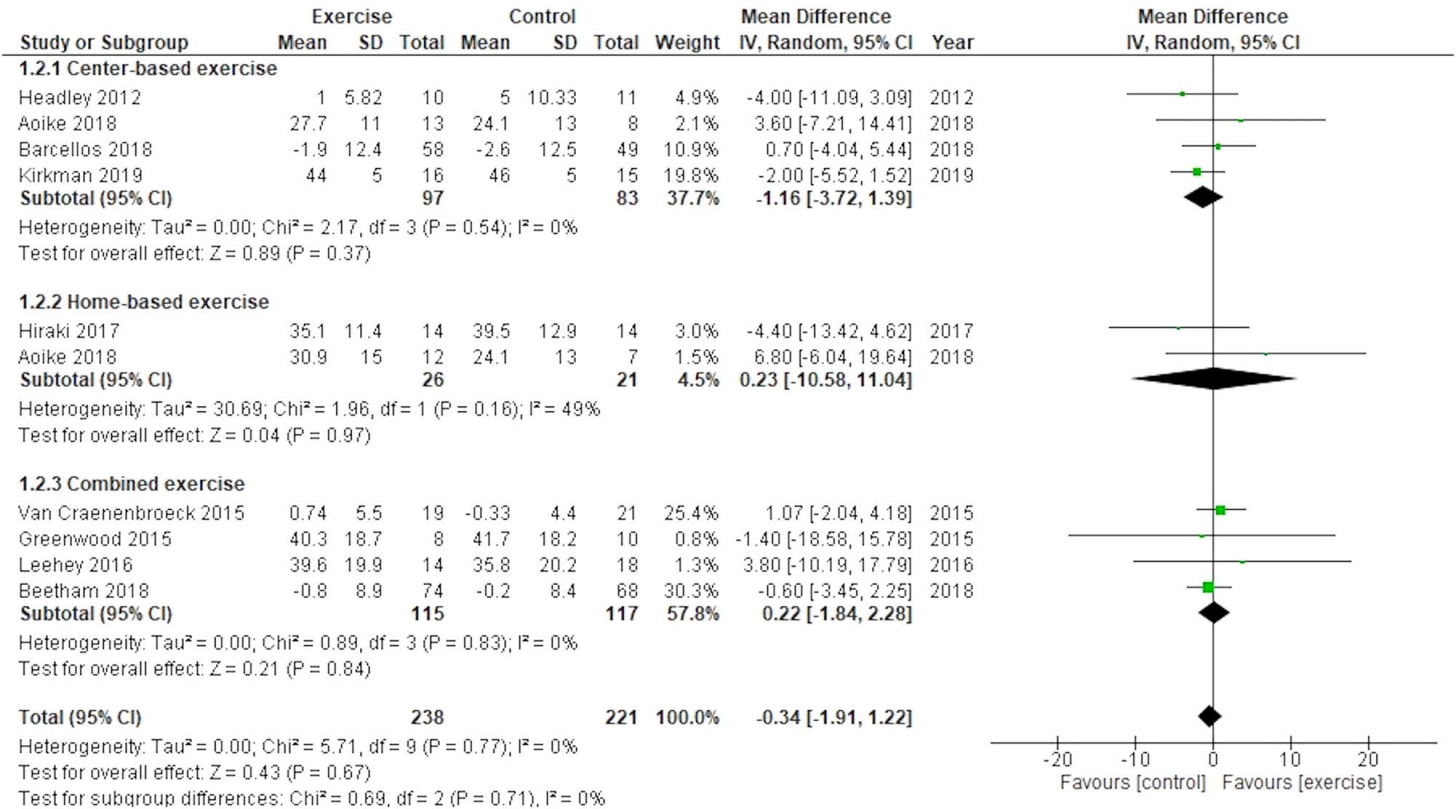
Effect of exercise training on estimated glomerular filtration rate.

Nine trials including 459 participants reported eGFR as outcomes. eGFRs were mostly evaluated using the CKD-Epidemiology Collaboration (EPI) creatinine equation^18,20,28^ or the Modification of Diet in Renal Disease (MDRD) formula^8,16,17,19,30,36^. The results showed that the effect of exercise training on eGFR was not significant compared to usual care (mean difference (MD), − 0.34; 95% CI − 1.91 to 1.22; participants = 459, trials = 9; I^2^ = 0%) (Fig. 3). Subgroup analysis performed for exercise type, eGFR (< 30 or ≥ 30 mL/min/1.73 m^2^), and BMI (< 30 or ≥ 30 kg/m^2^) showed no evidence of differences between the groups (test for subgroup difference: P = 0.88, 0.19, and 0.58, respectively). Subgroup analysis was not performed for other variables because an I^2^ > 50% was not obtained.

Only 5 trials including 231 participants reported serum creatinine (Scr) (μmol/L) as outcomes. There was no evidence of effects of physical exercise interventions on Scr improvement compared to usual care (MD, 1.48; 95% CI − 7.50 to 1.31; participants = 231, trials = 10; I^2^ = 0%) (see Supplementary Fig. S1 online).

#### Physical capacity

**Figure 4:**
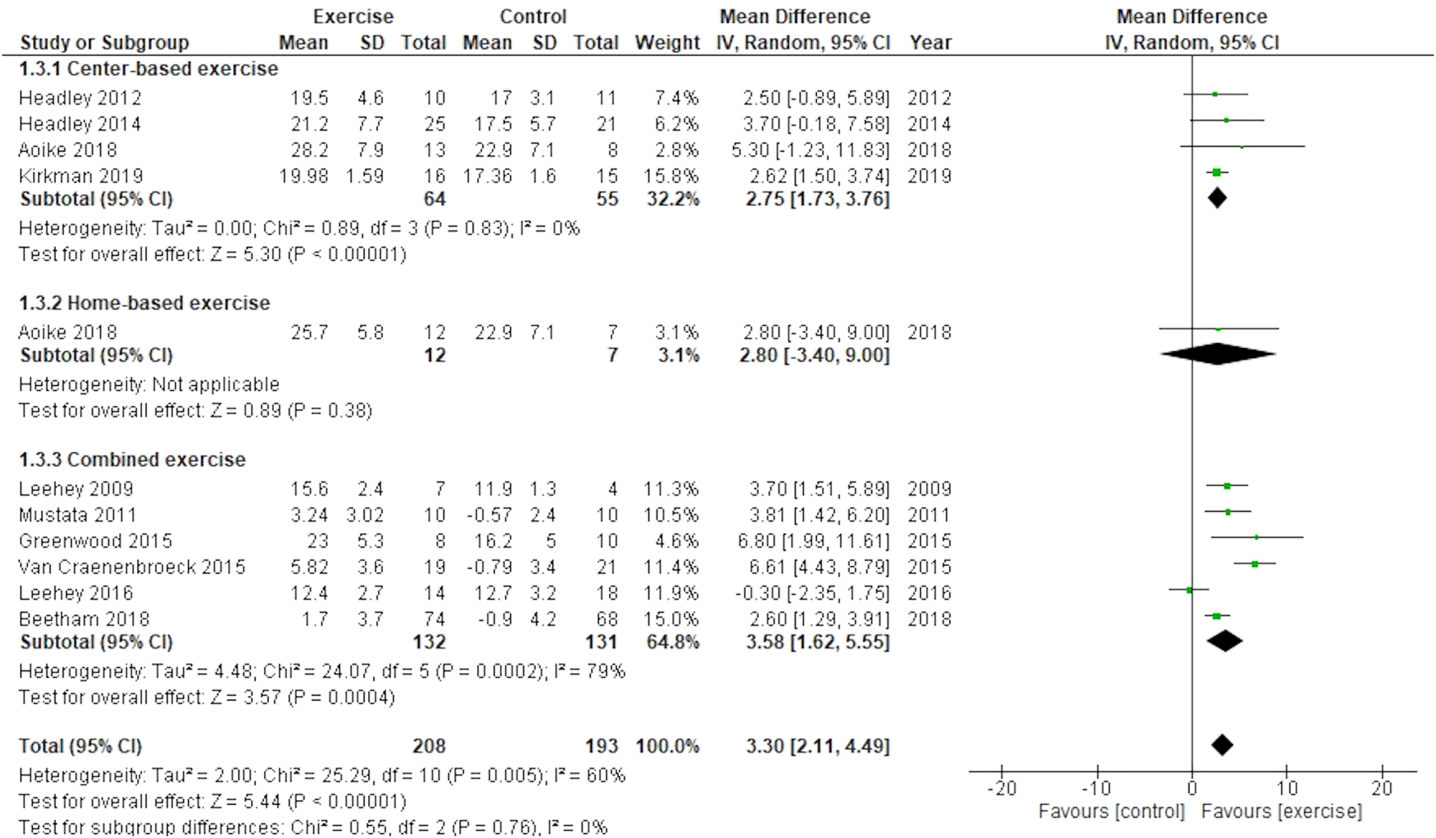
Effect of exercise training on peak/maximum oxygen uptakes.

Ten trials including 401 participants reported peak/maximum oxygen uptakes (peak/max VO_2_) as physical capacity outcomes. The results demonstrated significant improvements in the peak/max VO_2_ in the exercise group compared with the usual care group (MD, 3.30; 95% CI 2.11–4.49; participants = 401, trials = 10; I^2^ = 60%) (Fig. 4), although high heterogeneity was detected (P = 0.005, I^2^ = 60%). Subgroup analysis performed for each exercise type, eGFR (< 30 or ≥ 30 mL/min/1.73 m^2^), length of exercise intervention, and the percentage of patients with diabetes mellitus (DM) complications (≥ 50% or < 50%) showed no evidence of differences between the groups (test for subgroup difference: P = 0.66, 0.62, 0.69, and 0.37, respectively). Studies in which the basal average BMI of participants was < 30 kg/m^2^ (MD, 5.51; 95% CI 3.45–7.57; participants = 78, trials = 3; I^2^ = 37%) showed a more significant treatment effect than studies wherein the average BMI of participants was ≥ 30 kg/m^2^ (MD, 2.44; 95% CI 1.45–13.42; participants = 323, trials = 7; I^2^ = 37%); additionally, heterogeneity was lower in subgroup analysis than in pre-subgroup analysis (see Supplementary Table S1 online). In the meta-regression analyses of 9 trials (excluding one trial^39^ due inadequate BMI data), a significant association was observed between the MD of peak/max VO_2_ and BMI [slope:-0.555 BMI (95% CI − 0.925 to 0.186), P = 0.009] (see Supplementary Fig. S5 online). The length of exercise intervention and eGFR were not significantly associated with the MD of peak/max VO_2_ (P = 0.301 and 0.713, respectively).

#### Adverse events

Among the included records, 12 trials reported of adverse events related to exercise training; eleven trials showed no adverse event occurred during exercise training and tests. However, only 1 trial^21^ reported 11 adverse events possibly related to the study (6 cases of hypotension, 1 case of knee pain, 1 rapid atrial fibrillation case, 1 case of Achilles tendon pain, 1 case of joint pain, and 1 case of chest pain).

### Effects of interventions: secondary outcomes

#### Leg muscle strength

Only 4 trials, including 119 participants, reported leg muscle strength as a physical function outcome. Methods of evaluating leg muscle strength were different among the 4 trials (Hand-held dynamometer^8^, Biodex system 3 isokinetic testing system^16^, Cybex NORM Isokinetic Dynamometer^29^, Keiser resistance training equipment^41^). It was observed that the effect of exercise training on leg muscle strength in patients with non-dialysis CKD compared to usual care was uncertain (standard mean difference (SMD), 0.35; 95% CI − 0.03 to 0.73; participants = 119, trials = 4; I^2^ = 7%) (see Supplementary Fig. S2 online).

#### Six-minute walk distance

Five trials, including 392 participants, reported a 6-min walk distance as the walking capacity. Improvements in 6-min walk distance were observed in the exercise group compared to the usual care group (MD, 47.15; 95% CI 26.87–67.43; participants = 392, trials = 5; I^2^ = 64%) (see Supplementary Fig. S3 online), although high heterogeneity was detected.

#### Time of TUG

Only 3 trials including 170 participants reported timed up and go (TUG) results as dynamic balance evaluation. The results demonstrated that there were improvements with exercise training compared with usual care (MD, − 0.72; 95% CI − 1.21 to − 0.24; participants = 170, trials = 3; I^2^ = 0%) (see Supplementary Fig. S4 online).

#### Sensitivity analysis and publication bias

Sensitivity analysis was performed on the exclusion of studies for high risks of bias in the overall results, and there were no changes evident compared to the overall results. Funnel plots showed that trials evaluating eGFR were symmetrically distributed; on the contrary, the distribution of the plot in trials using peak/max VO_2_ was slightly asymmetrical (see Supplementary Figs. S6, S7 online). In Egger’s test, no significant publication bias was observed in trials using eGFR and peak/max VO_2_ (P = 0.955 and 0.261, respectively).

## Discussion

To the best of our knowledge, this is the first meta-analysis assessing the effects of physical exercise training on the risk of mortality, physical and kidney function, and adverse events exclusively in patients with non-dialysis CKD. The main findings of this review revealed that the effect of exercise training on all-cause mortality and kidney function could not be established in patients with non-dialysis CKD, while exercise training improved physical and walking capacity.

A previously reported systematic review which had conducted meta-analysis showed that exercise training significantly improved eGFR compared with usual care in patients with non-dialysis CKD ^43^. However, the review had included non-RCT studies^44,45^, and some study participants were included more than twice in the meta-analysis; this may have resulted in selection bias and the overestimation of the effect of exercise on kidney function. Some studies showed that exercise training improved vascular function, attenuated the increase in sympathetic nervous system activity, and reduced blood pressure in patients with non-dialysis CKD^17,18,28,33,35,38,44^. This supports the hypothesis that exercise training could delay the decline in kidney function. However, in our review, the effect of exercise training with moderate intensity on the rate of kidney function decline was found to be inconclusive. The duration of exercise intervention in the included trials may have been insufficient to demonstrate improvement in mortality rates and kidney function. As for non-RCT studies, a retrospective longitudinal cohort study^46^ reported that the completion of renal rehabilitation consisting of aerobic and resistance training for a 12-week period was associated with longer event-free survival during the follow-up period (median 34 months). Also, an observational study^47^ showed that substitution of sedentary activity with light activity, but not with exercise training, was associated with a lower hazard of death in the CKD group. Similarly, a previous study showed that muscle mass and physical activity affected SCr rather than cystatin C; thus, use of cystatin may be an adequate alternative to assess renal function^48^. Two trials^19,28^ included in our review measured cystatin; however, a meta-analysis could not be performed due to the limited number of trials. A future study assessing the effect of longer duration exercise training on mortality and kidney function based on cystatin levels is required. An increase in adverse events in the exercise training group compared with the usual care group could not be determined. Of all studies in this review, only one reported adverse event related to exercise training^21^. Therefore, a meta-analysis of adverse events could not be conducted; there may have been low rates of adverse events in exercise training overall.

Regular exercise training improved physical and walking capacity in patients with non-dialysis CKD; this was consistent with the results of a previous systematic review of the effect of exercise training in patients with CKD, including dialysis patients, kidney transplant patients, and heart failure patients^13,49–51^. Common symptoms of these chronic diseases (CKD including non-dialysis, dialysis, and kidney transplant patients, DM, and heart failure) were loss of muscle strength, lack of physical activity, and reduced physical capacity. A previous study found that physical capacity (e.g., peak/max VO_2_) was related to the mortality of patients with CKD^52^, suggesting that exercise training increased physical capacity and benefited patients with CKD. The results showed that lower BMI at baseline predicted greater improvements in peak/max VO_2_, while other factors were not significantly associated with the effect of exercise training.

In a previous study that included patients with heart failure, BMI was not associated with an improvement in physical capacity^53^, which is in contrast with our study result. However, explaining the association between BMI and improvement in physical capacity was difficult because only univariate meta-regression analysis was performed in this study due to the small trial sample size. This limits a concurrent consideration of the influence of other factors. Furthermore, adherence to exercise training was reported in only 67% of trials. Adherence rate for exercise training ranged from 63% to 96.9%, possibly affecting its influence on peak/max VO_2_. Further studies should assess the relationships between the effect of peak/max VO_2_ and other factors.

Leg muscle strength is an important marker of physical function that predicts mortality in patients with CKD receiving dialysis^54^. However, the effect of resistance exercise training on leg muscle strength was not significant because of the small number of trials involving patients with non-dialysis CKD. Previous reviews showed that progressive resistance training significantly improved standardized muscular strength in patients with CKD on dialysis^49^. Further research is required to determine whether resistance training improves leg muscle strength in patients with non-dialysis CKD.

The generalizability of this review was limited by age and cause of kidney disease. CKD is more common in people aged 65 or more years^1,55^, and diabetes and high blood pressure have been considered as causes of kidney disease^6,56,57^. However, the approximate mean age of participants in the included trials ranged from 50 to 65 years. The number of older adults may increase in the future; therefore, further studies should assess the effect of exercise training on elderly patients with CKD.

There are some limitations to this review. First, complete data were not obtained because there were missing data in some trials, despite efforts in reaching out to the authors. For this reason, there is a possibility of presence of predicted direction of bias. Secondly, some trials have a high risk of bias, especially those related to the predicted direction of bias, because of missing outcome data and information bias due to the absence of blinding. More high-quality RCTs are needed to clarify the effects of exercise training. Trials included in our review were mostly studies with short durations of intervention and follow-up periods were less than 1 year. Thus, the duration of exercise intervention may have been insufficient to show an improvement in the mortality rates and a significant association with exercise training in the meta-regression analysis. Our systematic review did not include non-RCTs, because RCTs are more likely to provide unbiased information about the differential effects of alternative health interventions (clearly defined exercise training or usual care) than non-RCTs. Therefore, inclusion of non-RCTs of good quality with longer follow-up periods could potentially alter the results. Finally, adherence to exercise training was not reported in 33% of trials, and this may have biased the effect of exercise on kidney and physical functions.

## Conclusion

Regular aerobic and/or resistance training improves physical and walking capacity for patients with non-dialysis CKD. The effect on mortality, kidney function, and leg muscle strength is inconclusive. Furthermore, few adverse events related to exercise training were reported, suggesting that regular exercise training with moderate intensity for 8 weeks to 1.5 years may be safe for patients with non-dialysis CKD. Future studies and multi-center RCTs with larger sample sizes and cohorts of elderly people are needed to focus on the effect of resistance training in non-dialysis CKD.

## Methods

### Protocol and registration

The protocol was registered on UMIN Clinical Trial Registry (UMIN ID000039799). The meta-analysis was performed following the Preferred Reporting Items for Systematic Reviews and Meta-Analysis (PRISMA) statement^58,59^. A systematic review was conducted in agreement with the recommendations stated in the Cochrane Handbook^60^.

### Eligibility criteria

#### Types of study

We included all RCTs, cluster-RCTs, and cross-over trials that investigated the effects of physical exercise interventions on physical function, kidney disease, and mortality of patients with non-dialysis CKD. RCTs without appropriate control groups, including those lacking usual care treatment arms, were excluded. Similarly, quasi-RCTs were excluded because their allocation of participants to treatments is not randomized.

#### Participants

Adult participants older than 18 years of age who were diagnosed with CKD were excluded. CKD was defined according to the Clinical Practice Guideline for the Evaluation and Management of Chronic Kidney Disease^2^. CKD was defined based on the following criteria: (1) abnormal kidney structure or function and (2) presence of CKD for more than 3 months with impaired health status. Abnormal kidney function was defined as decreased GFR (< 60 mL/min/1.73 m^2^) or detection of one or more abnormalities for markers of kidney damage, such as (1) albuminuria (albumin excretion rate (AER) > 30 mg/24 h; albumin creatinine ratio (ACR) > 30 mg/g [> 3 mg/mmol]), (2) urine sediment abnormalities, (3) electrolyte imbalance and other abnormalities due to tubular disorders, (4) histological abnormalities, (5) structural abnormalities revealed by imaging exams, and (6) history of kidney transplantation^2^. Participants who have undergone renal replacement therapies, such as dialysis or kidney transplant, were excluded.

#### Types of interventions

Hospital-based or home-based exercise interventions were included if supervised by health professionals or self-training. Similarly, different types of exercise, such as resistance training, aerobic exercise, or both, were included. The interventions were compared to control interventions, such as usual care or no-exercise care, consisting of medical care. Studies with exercise interventions clearly defined for frequency (at least once a week), intensity (using percentage of peak workload/oxygen uptakes, anaerobic threshold, or Borg scales), or duration of exercise (more than one month) were included. Abnormal types of exercise were equally included.

### Outcome measures

The primary outcomes were as follows: (1) all-cause mortality; (2) kidney function (eGFR, Scr); (3) physical capacity (peak/max VO_2_); and (4) adverse events. On the contrary, the secondary outcomes were as follows: (1) muscle strength (leg muscle strength); (2) walking capacity (6-min walk distance); and (3) balance outcome (time of TUG test).

### Search strategy for the identification of relevant studies

On 16 August 2019, the PubMed, CINAHL, Cochrane Library databases, and Embase were electronically searched for eligible RCTs with no restrictions for date/time, language, document type, or publication status. A search strategy was adapted for use in the course of exploring the aforementioned databases (Online Appendix 1).

### Screening the studies

Two authors (KN, TS) independently screened all titles and abstracts for all potential studies against the inclusion criteria. Full reports were obtained for all titles that appeared to meet the inclusion criteria and for those wherein any uncertainty was observed. Subsequently, the two authors screened the full-text reports to determine whether these articles met the inclusion criteria. Reasons for the exclusion of ineligible studies were identified. In case of disagreements, a third reviewer (SY) provided comments and made a final decision. The entire screening process was recorded, and the study selection process is described in the PRISMA flow chart (Fig. 1).

### Data extraction

The two reviewers conducted the data extraction from eligible articles according to the recommendations stated in the PRISMA statement^58,59^. Disagreements were resolved by the third reviewer. Study characteristics and clinical outcome measures were extracted. The extracted data included general information (authors, year of publication, location), participant characteristics (sample size, inclusion/exclusion criteria, randomization process and allocation, mean age, gender, and percentage of patients with diabetes), interventions (the type of intervention, intensity, duration, and frequency), outcome measures (all-cause mortality, kidney function, including eGFR and Scr, physical function markers, including peak VO_2_, muscular leg strength, time of TUG, and 6-min walk distance). The corresponding authors of the included publications were contacted for missing data and further information if considered necessary.

### Risk of bias

The risk of bias was assessed using the Revised Cochrane risk-of-bias tool for randomized trials (RoB 2) criteria recommended by the Cochrane Handbook of Systematic Reviews of Interventions^60^. The domains for risk of bias are (1) randomization process, (2) deviations from intended interventions, (3) missing outcome data, (4) measurement of outcome data and (5) selection of the reported results. The risk of bias was categorized as low, some concerns, or high. After the judgment of all domains, the overall risk of bias was assessed by the judgment of all domains as low, some concerns, or high risk of bias. The two reviewers independently conducted the risk assessment, and the third reviewer resolved disagreements.

### Data synthesis strategy

Statistical analysis was performed using the Review Manager Software (RevMan V.5.3) to combine and calculate the effect size for each outcome according to the recommendations set by the statistical guidelines described in the Cochrane Handbook of Systematic Reviews of Interventions^60^. Meta-analyses of the data were performed if eligible studies were sufficiently clinical and statistically homogeneous. Clinical heterogeneity was assessed by considering the between-study variability for specific factors such as age or type of exercise interventions. Statistical heterogeneity was tested using the Chi-square test and the I^2^ statistic. In the event there was substantial heterogeneity between studies (I^2^ > 50% or P < 0.1), the study design and characteristics of the studies were examined. The possible causes of heterogeneity were explored by conducting sub-group, meta-regression, or sensitivity analyses. Random effect models were applied when appropriate. A meta-analysis was conducted if data were appropriate. Dichotomous data (mortality) were described using risk ratios (RR) with a 95% confidence interval (CI). Continuous outcomes were analyzed using weighted mean differences (WMD) (with 95% CI) or SMD (95% CI) if different measurement scales were used.

### Subgroup, meta-regression, or sensitivity analyses

Sub-group analysis was performed on all primary outcomes as follows: exercise types (center-based or home-based exercise, or a combination of center- and home-based exercise), basal eGFR (< 30 mL/min/1.73 m^2^ or ≥ 30 mL/min/1.73 m^2^), and basal BMI (< 30 kg/m^2^ or ≥ 30 kg/m^2^). For center-based exercises, patients participated in exercise training sessions under the real-time supervision of professionals either in a hospital or training center. For home-based exercises, patients performed exercise training at home or in a community setting without real-time supervision of professionals.

Furthermore, sub-group and meta-regression analyses were performed to explore the causes of heterogeneity among primary outcomes if an I^2^ > 50% was obtained. Meta-regression analysis was performed using the Stata 14 software (www.stata.com). The length of exercise intervention (< 24 weeks, 24–48 weeks, or ≥ 48 weeks) and percentage of patients with DM complications (≥ 50% or < 50%) were used as subgroup factors. In the univariate meta-regression model, eGFR, BMI, or length of intervention were used as independent factors.

Sensitivity analysis was performed to explore the sources of heterogeneity, such as the exclusion of studies with a high risk of bias and the evaluation of meaningful changes in the effect size.

### Assessment of publication bias

The potential for publication bias was assessed using funnel plots and Egger`s test if more than ten studies were available.

## Supplementary information

Supplementary Information

## Data Availability

We confirm that the data supporting the findings of this review are available within the article and its supplementary materials.
