# Supplementary material for: Effects of exercise on kidney and physical function in patients with non-dialysis chronic kidney disease: a systematic review and meta-analysis
Source: Sci Rep. 2020 Oct 23;10:18195. doi: 10.1038/s41598-020-75405-x (PMC7585437; doi:10.1038/s41598-020-75405-x)
Supplement: Supplementary file 1 — Supplementary Information [file 41598_2020_75405_MOESM1_ESM.docx]

**Effects of exercise on kidney and physical function in patients with non-dialysis chronic kidney disease: a systematic review and meta-analysis**

**Keisuke Nakamura^1, 6*^, Tomohiro Sasaki^1^, Shuhei Yamamoto^2^, Hiroto Hayashi^3^, Shinji Akou^4^ & Yuu Tanaka^5^**

^1^ Department of Rehabilitation, Matsumoto City Hospital, Nagano, Japan

^2^ Department of Rehabilitation, Shinshu University Hospital, Nagano, Japan

^3^ Department of Public Health, Graduate School of Medical and Dental Sciences, Tokyo Medical and Dental University, Tokyo, Japan

^4^ Department of Internal Medicine, Matsumoto City Hospital, Nagano, Japan

^5^ Department of Anesthesiology, Nara Medical University, Nara, Japan

^6^ Present address: Department of Rehabilitation, Matsumoto City Hospital, 4417-180 Hata, Matsumoto, Nagano 390-1401, Japan

**^*^** Email: keipons55@yahoo.co.jp

**Supplemental data**


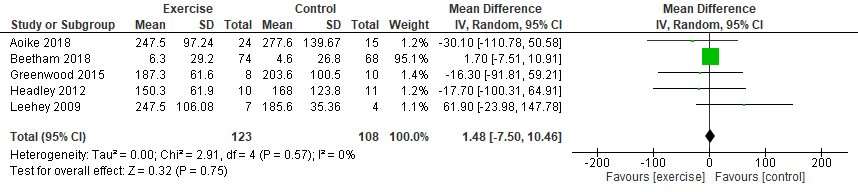


**Supplementary Figure S1. Effect of exercise training on serum creatinine.**


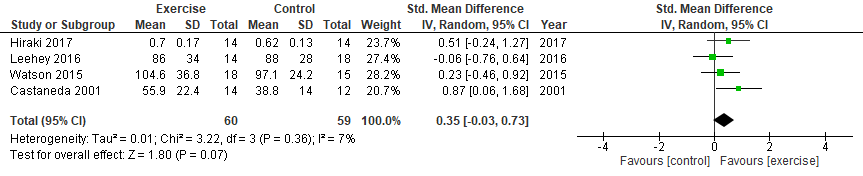


**Supplementary Figure S2. Effect of exercise training on leg muscle strength.**


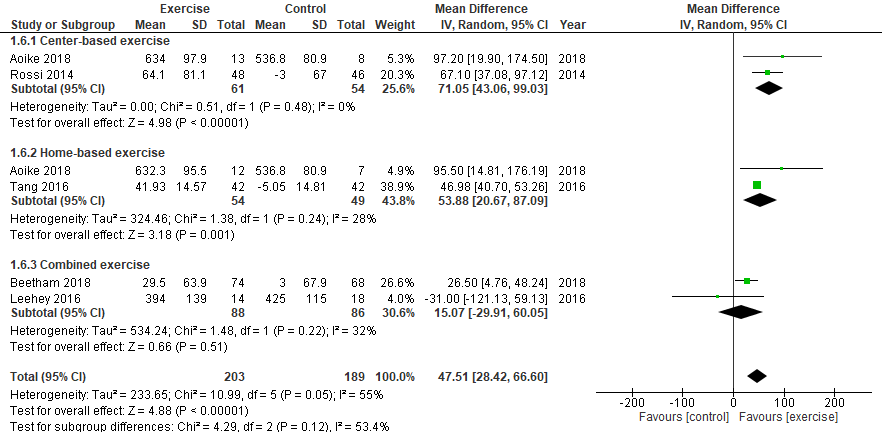


**Supplementary Figure S3. Effect of exercise training on 6 minute walk distance.**


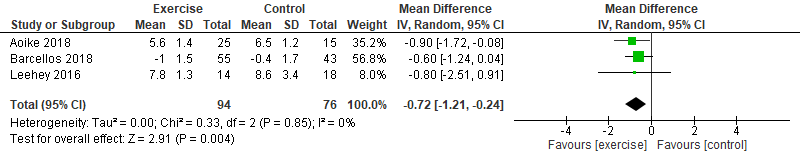


**Supplementary Figure S4. Effect of exercise training on time of TUG.**

**Supplementary Table. Subgroup analysis**

| Outcome | Subgroups | Included studies | Heterogeneity | MD (95%CI) | Test for subgroup differences |
| --- | --- | --- | --- | --- | --- |
| Peak/max VO_2_ | eGFR < 30 (ml/min/1.73m^2^) | Aoike 2018; Mustata 2011 | Tau^2^ = 0.00 I^2^ = 0% | 3.87  (1.76, 5.98) | Chi^2^ = 0.25, df = 1 (p=0.62), I^2^ = 0% |
|  | eGFR ≧30 (ml/min/1.73m^2^) | Kirkman 2019; Beetham 2018; Leehey 2016, 2009; Greenwood 2015; Van Craenenbroeck 2015; Headley 2014, 2012 | Tau^2^ = 2.60 I^2^ = 71% | 3.22 (1.81, 4.64) |  |
|  | BMI < 30 (kg/m^2^) | Greenwood 2015; Van Craenenbroeck 2015; Mustata 2011 | Tau^2^ = 1.25 I^2^ = 37% | 5.51  (3.45, 7.57) | Chi^2^ = 6.96, df = 1 (p=0.008), I^2^ = 85.6% |
|  | BMI ≧30 (kg/m^2^) | Aoike 2018; Kirkman 2019; Beetham 2018; Leehey 2016, 2009; Headley 2014, 2012 | Tau^2^ = 0.57 I^2^ = 35% | 2.44 (1.45, 13.42) |  |
|  | DM < 50% | Aoike 2018; Kirkman 2019; Beetham 2018; Greenwood 2015; Van Craenenbroeck 2015; Headley 2014, 2012 Mustata 2011 | Tau^2^ = 1.59 I^2^ = 64% | 3.31  (2.10, 4.52) | Chi^2^ = 0.81, df = 1 (p=0.37), I^2^ = 0% |
|  | DM ≧50% | Leehey 2016, 2009; Headley 2014, 2012; Mustata 2011 | Tau^2^ = 4.60 I^2^ = 78% | 2.36 (-0.38, 5.10) |  |
|  | < 24 weeks | Headley 2014, 2012 | Tau^2^ = 3.45 I^2^ = 71% | 3.89 (1.66, 6.13) | Chi2 = 0.74, df = 2 (p=0.69), I^2^ = 0% |
|  | 24-48 weeks | Aoike 2018; Leehey 2009 | Tau^2^ = 0.57 I^2^ = 37% | 3.78 (1.81, 5.74) |  |
|  | 48 weeks < | Kirkman 2019; Beetham 2018; Leehey 2016; Greenwood 2015; Van Craenenbroeck 2015; Mustata 2011 | Tau^2^ = 0.57 I^2^ = 38% | 2.66 (0.45, 4.87) |  |
| BMI; body mass index. VO_2_: oxygen uptakes. eGFR; estimated glomerular filtration rate | | | | | |


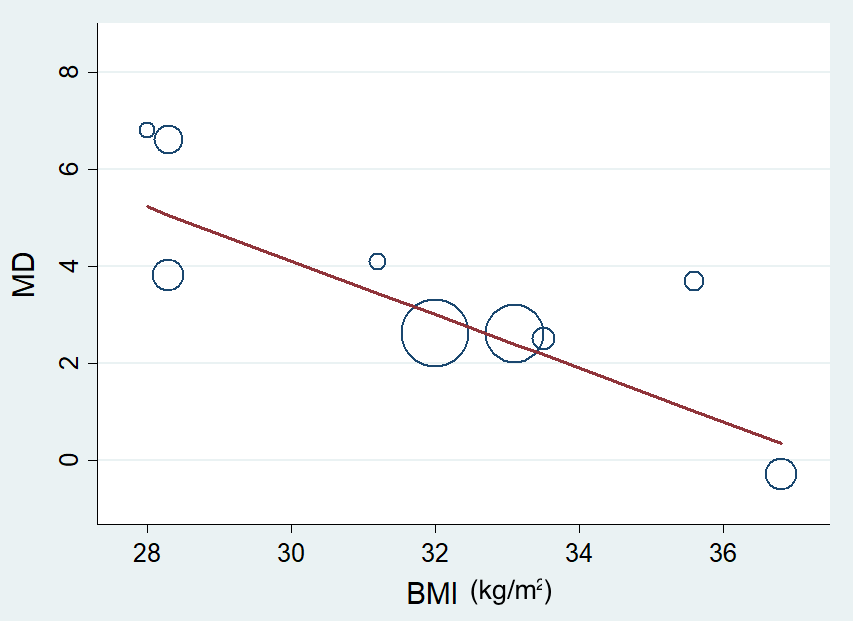


**Supplementary Figure S5. Bubble plot of BMI vs mean difference of peak/maximum oxygen uptakes. The size of each bubble is proportional to the weight assigned to study.**


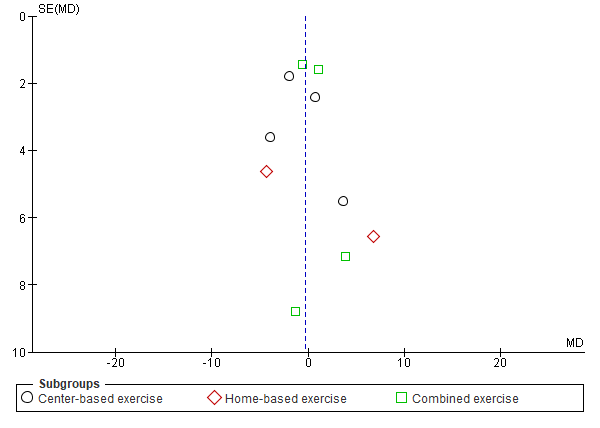


**Supplementary Figure S6. Funnel plot of between-groups analysis for eGFR.**


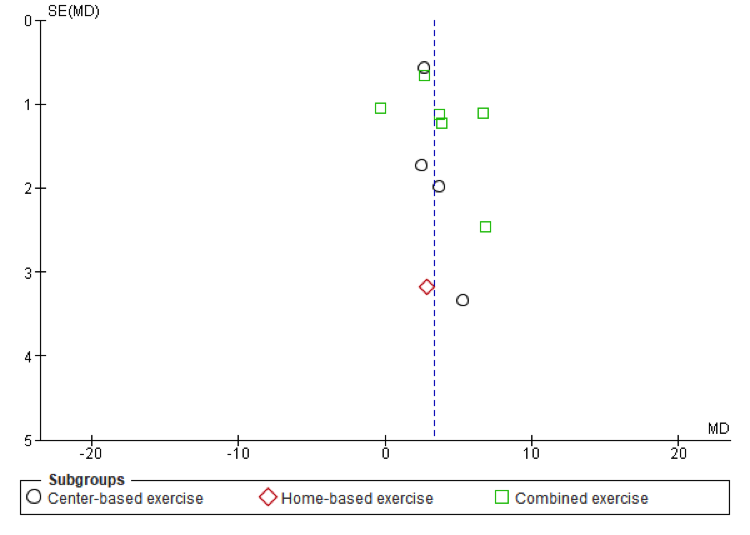


**Supplementary Figure S7. Funnel plot of between-groups analysis for peak/maximum oxygen uptakes.**

**Appendix**

**Search strategy**

**PubMed**

#1 Search "Exercise Therapy"[mh] OR "Exercise"[mh] OR "Exercise Test"[mh] OR

"Physical Education and Training"[mh] OR "Physical Fitness"[mh]

#2 Search exercise*[tiab] OR exertion*[tiab]

#3 Search physical education*[tiab] OR physical training*[tiab] OR physical fitness*[tiab]

OR physical rehabilitation*[tiab]

#4 Search resistance training*[tiab] OR resistance program*[tiab]

#5 Search muscle strength*[tiab]

#6 Search strength program*[tiab] OR strength training*[tiab]

#7 Search #1 or #2 or #3 or #4 or #5 or #6

#8 Search "Kidney Diseases"[mh] OR "Proteinuria"[mh]

#9 Search chronic kidney disease*[tiab] OR chronic renal disease*[tiab] OR chronic kidney

insufficienc*[tiab] OR chronic renal insufficienc*[tiab] OR chronic kidney failure*[tiab]

OR chronic renal failure*[tiab]

#10 Search uremi*[tiab] OR uraemi*[tiab] OR albuminuria*[tiab] OR proteinuria*[tiab] OR

urinary albumin*[tiab] OR urine albumin*[tiab] OR urinary protein*[tiab] OR urine

protein*[tiab]

#11 Search kidney disease*[tiab] OR nephropath*[tiab]

#12 Search #8 or #9 or #10 or #11

#13 Search kidney transplant*[ti] OR renal transplant*[ti] OR kidney graft*[ti] OR renal

graft*[ti]

#14 Search #12 not #13

#15 Search #7 and #14

#16 Search randomized controlled trial[pt] OR controlled clinical trial[pt] OR

randomized[tiab] OR placebo[tiab] OR drug therapy[sh] OR randomly[tiab] OR

trial[tiab] OR groups[tiab]

#17 Search animals[mh] NOT humans[mh]

#18 Search #16 not #17

#19 Search #15 and #18

**CINAHL**

S1 (MH "Therapeutic Exercise+") OR (MH "Exercise Therapy: Muscle Control (Iowa

NIC)") OR (MH "Exercise Therapy: Joint Mobility (Iowa NIC)") OR (MH "Exercise

Therapy: Balance (Iowa NIC)") OR (MH "Exercise Therapy: Ambulation (Iowa NIC)")

S2 (MH "Exercise+") OR (MH "Resistance Training") OR (MH "Exercise Test,

Cardiopulmonary") OR (MH "Exercise Test, Muscular+") OR (MH "Warm-Up

Exercise") OR (MH "Recovery, Exercise") OR (MH "Group Exercise") OR (MH

"Exercise Test+") OR (MH "Exercise Intensity") OR (MH "Kegel Exercises") OR (MH

"Open Kinetic Chain Exercises")

S3 (MH "Physical Education and Training+") OR (MH "Balance Training, Physical") OR

(MH "Education, Physical Therapy") OR (MH "Physical Education, Adapted") OR (MH

"Education, Physical Education") OR (MH "Education, Athletic Training")

S4 (MH "Physical Fitness+")

S5 TI ( exercise* OR exertion* ) OR AB ( exercise* OR exertion* )

S6 TI ( physical W2 (education* OR training* OR fitness* OR rehabilitation*) ) OR AB

( physical W2 (education* OR training* OR fitness* OR rehabilitation*) )

S7 TI ( resistance W2 (training* OR program*) ) OR AB ( resistance W2 (training* OR

program*) )

S8 TI ( strength* N3 (muscle* OR program* OR training*) ) OR AB ( strength* N3

(muscle* OR program* OR training*) )

S9 S1 OR S2 OR S3 OR S4 OR S5 OR S6 OR S7 OR S8

S10 (MH "Kidney Diseases+")

S11 (MH "Proteinuria+")

S12 TI ( chronic W2 (kidney OR renal) W2 (disease* OR insufficienc* OR failure*) ) OR

AB ( chronic W2 (kidney OR renal) W2 (disease* OR insufficienc* OR failure*) )

S13 TI ( uremi* OR uraemi* OR albuminuria* OR proteinuria* OR (urin* N2 (albumin* OR

protein*)) ) OR AB ( uremi* OR uraemi* OR albuminuria* OR proteinuria* OR (urin*

N2 (albumin* OR protein*)) )

S14 TI ( (kidney W2 disease*) OR nephropath* ) OR AB ( (kidney W2 disease*) OR

nephropath* )

S15 S10 OR S11 OR S12 OR S13 OR S14

S16 TI (kidney OR renal) N3 (transplant* OR graft*)

S17 TI S15 NOT S16

S18 S9 AND S17

S19 PT CLINICAL TRIAL

S20 (MH "Randomized Controlled Trials+") OR (MH "Clinical Trials+")

S21 TI ( randomized OR randomly OR trial OR placebo OR groups ) OR AB ( randomized

OR randomly OR placebo OR groups )

S22 S19 OR S20 OR S21

S23 (MH "Human")

S24 S22 AND S23

S25 S18 AND S24

**The Cochrane Library databases**

#1 [mh "Exercise Therapy"] OR [mh "Exercise"] OR [mh "Exercise Test"] OR [mh

"Physical Education and Training"] OR [mh "Physical Fitness"]

#2 (exercise* OR exertion*):ti,ab,kw

#3 (physical NEXT (education* OR training* OR fitness* OR rehabilitation*)):ti,ab,kw

#4 (resistance NEXT (training* OR program*)):ti,ab,kw

#5 (strength* NEAR/3 (muscle* OR program* OR training*)):ti,ab,kw

#6 #1 or #2 or #3 or #4 or #5

#7 [mh "Kidney Diseases"] OR [mh "Proteinuria"]

#8 (chronic NEXT (kidney OR renal) NEXT (disease* OR insufficienc* OR

failure*)):ti,ab,kw

#9 (uremi* OR uraemi* OR albuminuria* OR proteinuria* OR (urin* NEAR/2 (albumin*

OR protein*))):ti,ab,kw

#10 ((kidney NEXT disease*) OR nephropath*):ti,ab,kw

#11 #7 or #8 or #9 or #10

#12 ((kidney OR renal) NEAR/3 (transplant* OR graft*)):ti

#13 #11 not #12

#14 #6 and #13

#15 #6 and #13 in Cochrane Reviews, Cochrane Protocols

#16 #6 and #13 in Trials

**Embase**

S1 (TI,AB(exercise* OR exertion*))

S2 (TI,AB(physical P/2 (education* OR training* OR fitness* OR rehabilitation*)))

S3 (TI,AB(resistance P/2 (training* OR program*)))

S4 (TI,AB(strength* N/3 (muscle* OR program* OR training*)))

S5 (EMB.EXACT.EXPLODE("kinesiotherapy") OR EMB.EXACT.EXPLODE("exercise")

OR EMB.EXACT.EXPLODE("exercise test"))

S6 (EMB.EXACT.EXPLODE("physical education") OR

EMB.EXACT.EXPLODE("fitness"))

S7 S1 OR S2 OR S3 OR S4 OR S5 OR S6

S8 (EMB.EXACT.EXPLODE("kidney disease")) OR

EMB.EXACT.EXPLODE("proteinuria")

S9 (TI,AB(chronic P/2 (kidney OR renal) P/2 (disease* OR insufficienc* OR failure*)))

S10 (TI,AB(uremi* OR uraemi* OR albuminuria* OR proteinuria* OR (urin* N/2 (albumin*

OR protein*))))

S11 (TI,AB((kidney P/2 disease*) OR nephropath*))

S12 S8 OR S9 OR S10 OR S11

S13 (TI((kidney OR renal) N/3 (transplant* OR graft*)))

S14 (S12 NOT S13)

S15 (S7 AND S14)

S16 ((EMB.EXACT("controlled clinical trial") OR EMB.EXACT.EXPLODE("clinical trial

(topic)") OR EMB.EXACT("randomized controlled trial")) OR (TI,AB(randomized) OR

TI,AB(randomly) OR TI(trial) OR TI,AB(placebo) OR TI,AB(groups)))

S17 (ANIMAL(YES) NOT HUMAN(YES))

S18 (S16 NOT S17)

S19 (S15 AND S18)
